# Diagnosis of temporomandibular disorders using artificial intelligence technologies: A systematic review and meta-analysis

**DOI:** 10.1371/journal.pone.0272715

**Published:** 2022-08-18

**Authors:** Nayansi Jha, Kwang-sig Lee, Yoon-Ji Kim

**Affiliations:** 1 University of Ulsan College of Medicine, Seoul, Korea; 2 AI Center, Korea University Anam Hospital, Korea University College of Medicine, Seoul, Korea; 3 Department of Orthodontics, Asan Medical Center, University of Ulsan College of Medicine, Seoul, Korea; Thamar University, Faculty of Dentistry, YEMEN

## Abstract

**Background:**

Artificial intelligence (AI) algorithms have been applied to diagnose temporomandibular disorders (TMDs). However, studies have used different patient selection criteria, disease subtypes, input data, and outcome measures. Resultantly, the performance of the AI models varies.

**Objective:**

This study aimed to systematically summarize the current literature on the application of AI technologies for diagnosis of different TMD subtypes, evaluate the quality of these studies, and assess the diagnostic accuracy of existing AI models.

**Materials and methods:**

The study protocol was carried out based on the preferred reporting items for systematic review and meta-analysis protocols (PRISMA). The PubMed, Embase, and Web of Science databases were searched to find relevant articles from database inception to June 2022. Studies that used AI algorithms to diagnose at least one subtype of TMD and those that assessed the performance of AI algorithms were included. We excluded studies on orofacial pain that were not directly related to the TMD, such as studies on atypical facial pain and neuropathic pain, editorials, book chapters, and excerpts without detailed empirical data. The risk of bias was assessed using the QUADAS-2 tool. We used Grading of Recommendations, Assessment, Development, and Evaluations (GRADE) to provide certainty of evidence.

**Results:**

A total of 17 articles for automated diagnosis of masticatory muscle disorders, TMJ osteoarthrosis, internal derangement, and disc perforation were included; they were retrospective studies, case-control studies, cohort studies, and a pilot study. Seven studies were subjected to a meta-analysis for diagnostic accuracy. According to the GRADE, the certainty of evidence was very low. The performance of the AI models had accuracy and specificity ranging from 84% to 99.9% and 73% to 100%, respectively. The pooled accuracy was 0.91 (95% CI 0.76–0.99), I^2^ = 97% (95% CI 0.96–0.98), *p* < 0.001.

**Conclusions:**

Various AI algorithms developed for diagnosing TMDs may provide additional clinical expertise to increase diagnostic accuracy. However, it should be noted that a high risk of bias was present in the included studies. Also, certainty of evidence was very low. Future research of higher quality is strongly recommended.

## Introduction

Temporomandibular disorders (TMDs) can cause pain and dysfunction in the temporomandibular joints (TMJs) and masticatory muscles. TMDs are the second most common musculoskeletal conditions and include various symptoms, such as decreased range of motion, joint sound, and mouth opening deviation [1]. TMDs can be classified as pain-related disorders, which include myalgia and arthralgia, and intra-articular disorders, which include internal derangement and degenerative joint disease (DJD) [2].

The etiology of TMDs is considered multifactorial, with biological, behavioral, and psychosocial factors contributing independently or as interrelated factors [3, 4]. Moreover, comorbidities, such as cardiovascular diseases, osteoarthritis, tinnitus, sinusitis, and thyroid disorders, are associated with disease onset and progression [5–7]. Therefore, diagnosis of TMDs requires a comprehensive evaluation of the patients’ signs and symptoms (acquired through clinical examination and medical image analysis) and behavioral and psychosocial factors [2, 8]. Subsequently, the complex nature of TMDs makes diagnosis difficult.

Currently, the most widely accepted diagnostic criteria is the Diagnostic Criteria for Temporomandibular Disorders (DC-TMD) [2] which was developed on the basis of large-scale international studies and data analyses since the 1990s. The DC-TMD comprises two axes, Axis I and Axis II, which include diagnostic standards for differentiating pain-related TMDs and intra-articular disorders (Axis I) and assessing jaw function and behavioral and psychosocial factors (Axis II).

Despite the popularity of the DC-TMD, it has limitations in terms of its diagnostic accuracy. Several subtypes of internal derangement, such as disc displacement with reduction, with reduction and locking, and without reduction, showed low sensitivity (0.34–0.54). Similarly, low sensitivity (0.55) and specificity (0.61) were observed for DJD. Further, the interexaminer reliability is relatively low for internal derangement and DJD [2]. Screening tools, such as surveys to determine patients’ symptoms, are expensive and time-consuming and place a burden on clinicians.

Advancements in artificial intelligence (AI) technologies have led to major developments in the healthcare industry. The Merriam–Webster dictionary defines AI as ‘the capability of a machine to imitate intelligent human behavior.’ It essentially refers to the simulation of human intelligence processes using computer systems. Generally, AI systems are trained using large amounts of input data. Patterns are learned from these data and then used to predict the outcome of new instances. AI algorithms are increasingly applied in patient diagnoses, especially for detecting and classifying lesions, such as skin cancers [9], diabetic retinopathy [10], brain tumors [11], and dental caries [12], using medical diagnostic images [13]. Additionally, other data types, such as electronic medical records in the form of text [14], voice [15], and sound [16] are used to develop diagnostic tools to support clinicians in decision-making.

Recently, various AI algorithms have been applied to image and nonimage data for TMDs diagnosis [17–21]. However, studies on the use of AI for TMD diagnosis have used different patient selection criteria, disease subtypes, input data used for diagnosis, and outcome measures for performance evaluation. Moreover, the accuracy of the AI models varies. To the best of our knowledge, there has been no systematic review till date that summarizes such findings. Therefore, this study aimed to systematically summarize the current literature on the application of AI technologies for diagnosis of different TMD subtypes—both muscular and articular conditions—evaluate the quality of these studies and assess the diagnostic accuracy of existing AI models.

## Materials and methods

This systematic review and meta-analysis was conducted and reported in accordance with the Preferred Reporting Items for Systematic Review and Meta-analysis (PRISMA) 2020 guidelines (S1 and S2 Tables) [22].

### Research questions

This systematic review and meta-analysis was conducted to answer the following question: “How accurate are the AI algorithms for the diagnosis of TMDs?” The focused question was further classified as follows:

1. Which data were used for developing algorithms for TMD diagnosis?
2. Which AI techniques were used for TMD diagnosis?
3. Which features were used for TMD diagnosis?
4. Which outcome measures were used for assessing the model performance?

Further, the research question was formatted using the Population, Intervention, Comparison, and Outcome framework (Table 1).

**Table 1:** Description of the population, intervention, comparison, and outcome elements.

Research question	How accurate are the AI algorithms for the diagnosis of TMDs?
**Population**	Patients with TMDs
**Intervention**	Use of medical diagnostic images (CBCT, MRI, panoramic radiographs) and health records
**Comparison**	Type of data and algorithm used for AI-based automated diagnosis models
**Outcome**	Performance of AI algorithms for the diagnosis of TMDs assessed using diagnostic accuracy

AI, artificial intelligence; TMDs, temporomandibular disorders; CBCT, cone-beam computed tomography; MRI, magnetic resonance imaging

### Information sources and search strategy

Our search algorithm comprised the PubMed, EMBASE, and Web of Science databases. A combination of the following terms was used: “artificial intelligence” OR “neural network” OR “machine learning” OR “deep learning” OR/AND “TMJ osteoarthritis” OR “temporomandibular joint osteoarthritis” OR “temporomandibular disorders” OR “masticatory muscle disorders” OR “TMDs” OR “TMJ disorder” OR “temporomandibular joint disorders” OR “TMJ arthritis” OR “temporomandibular joint arthritis” OR “progressive condylar resorption” OR “degenerative joint disease” OR “temporomandibular joint disease” OR “TMJ disease” OR “idiopathic condylar resorption” OR “juvenile idiopathic arthritis.” No start date was used, whereas the end date was June 30, 2022. Table 2 includes the search strategy for each database.

**Table 2:** Search strategy for each database.

Database	Search Terms	Records retrieved
PubMed	("artificial intelligence " OR " neural network " OR " machine learning " OR " deep learning ")) AND/OR (("TMJ osteoarthritis" OR "Temporomandibular joint osteoarthritis" OR " Temporomandibular disorders " OR "TMDs" OR "TMJ disorder" OR "Temporomandibular joint disorders" OR "TMJ arthritis" OR "Temporomandibular joint arthritis" OR "masticatory muscle disorder" OR "progressive condylar resorption" OR "degenerative joint disease" OR "Temporomandibular joint disease" OR "TMJ disease" OR "idiopathic condylar resorption" OR " juvenile idiopathic arthritis")	1142
Embase	("artificial intelligence " OR " neural network " OR " machine learning " OR " deep learning ")) AND/OR ((" TMJ osteoarthritis " OR " Temporomandibular joint osteoarthritis" OR " Temporomandibular disorders " OR " TMDs" OR " TMJ disorder" OR " Temporomandibular joint disorders" OR " TMJ arthritis" OR " Temporomandibular joint arthritis" OR "masticatory muscle disorder" OR "progressive condylar resorption" OR " degenerative joint disease" OR " Temporomandibular joint disease" OR " TMJ disease" OR "idiopathic condylar resorption" OR " juvenile idiopathic arthritis")	585
Web of Science	("artificial intelligence " OR " neural network " OR " machine learning " OR " deep learning ")) AND/OR ((" TMJ osteoarthritis " OR " Temporomandibular joint osteoarthritis" OR " Temporomandibular disorders " OR " TMDs" OR " TMJ disorder" OR " Temporomandibular joint disorders" OR " TMJ arthritis" OR " Temporomandibular joint arthritis" OR "masticatory muscle disorder" OR " progressive condylar resorption" OR " degenerative joint disease" OR " Temporomandibular joint disease" OR " TMJ disease" OR "idiopathic condylar resorption" OR " juvenile idiopathic arthritis")	196

### Eligibility criteria, study selection, and data collection

We included original studies published in scientific journals whose full texts were available. The inclusion criteria were as follows: (a) use of AI algorithms to diagnose at least one subtype of TMDs; (b) the performance of the developed AI algorithms was assessed; (c) no limit on the participants in terms of gender, age, or ethnicity; and (d) were written in English. The exclusion criteria were as follows: (a) studies on orofacial pain that is not directly related to the TMJ, such as atypical facial pain and neuropathic pain; (b) studies on TMJ that were unrelated to disease diagnosis; (c) editorials, comments, book chapters, and excerpts without detailed empirical data; and (d) studies not written in English.

To determine the final eligibility, the two investigators (YJK and NJ) independently assessed the full text of studies. Conflicts between the reviewers was resolved by the involvement of a third investigator (KSL). Then, two investigators, NJ and YJK, independently extracted and formulated the data, such as input data used for TMD diagnosis, AI algorithms used, and performance measures. Any discrepancies were resolved through discussion.

### Risk of bias assessment

The selected articles were critically assessed and scored independently by two investigators (YJK and NJ). Quality assessment of the studies was based on the Quality Assessment of Diagnostic Accuracy Studies (QUADAS-2) [23]. The QUADAS tool was first developed in 2003 for systematic reviews of diagnostic accuracy studies and later updated to QUADAS-2. It comprises four components: patient selection, index test, reference standard, and flow and timing. Each component is assessed for the risk of bias. The first three components are also assessed for concerns about the applicability of each component [23]. The quality was rated as high, low, or unclear. Conflicts between the reviewers was resolved by the involvement of a third investigator (KSL).

### Certainty of evidence assessment

We used Grading of Recommendations, Assessment, Development, and Evaluation (GRADE) [24] to evaluate the quality of evidence of studies for which meta-analysis was performed. Each outcome gets a rating on the quality of evidence of high, moderate, low, or very low within five domains- risk of bias, imprecision, inconsistency, indirectness, and publication bias.

### Statistical analysis

Meta-analysis of diagnostic accuracy was conducted using the Hartung–Knapp–Sidik–Jonkman method for random-effects models. The accuracy estimates were transformed using the Freeman–Tukey double arcsine method. Heterogeneity was quantified using the *I*^2^ statistic, which is the percentage of total variation across studies due to heterogeneity rather than chance. All analyses were conducted using R v.4.0.4 (R Project for Statistical Computing) with the Meta package.

## Results

### Study selection

**Fig 1:**
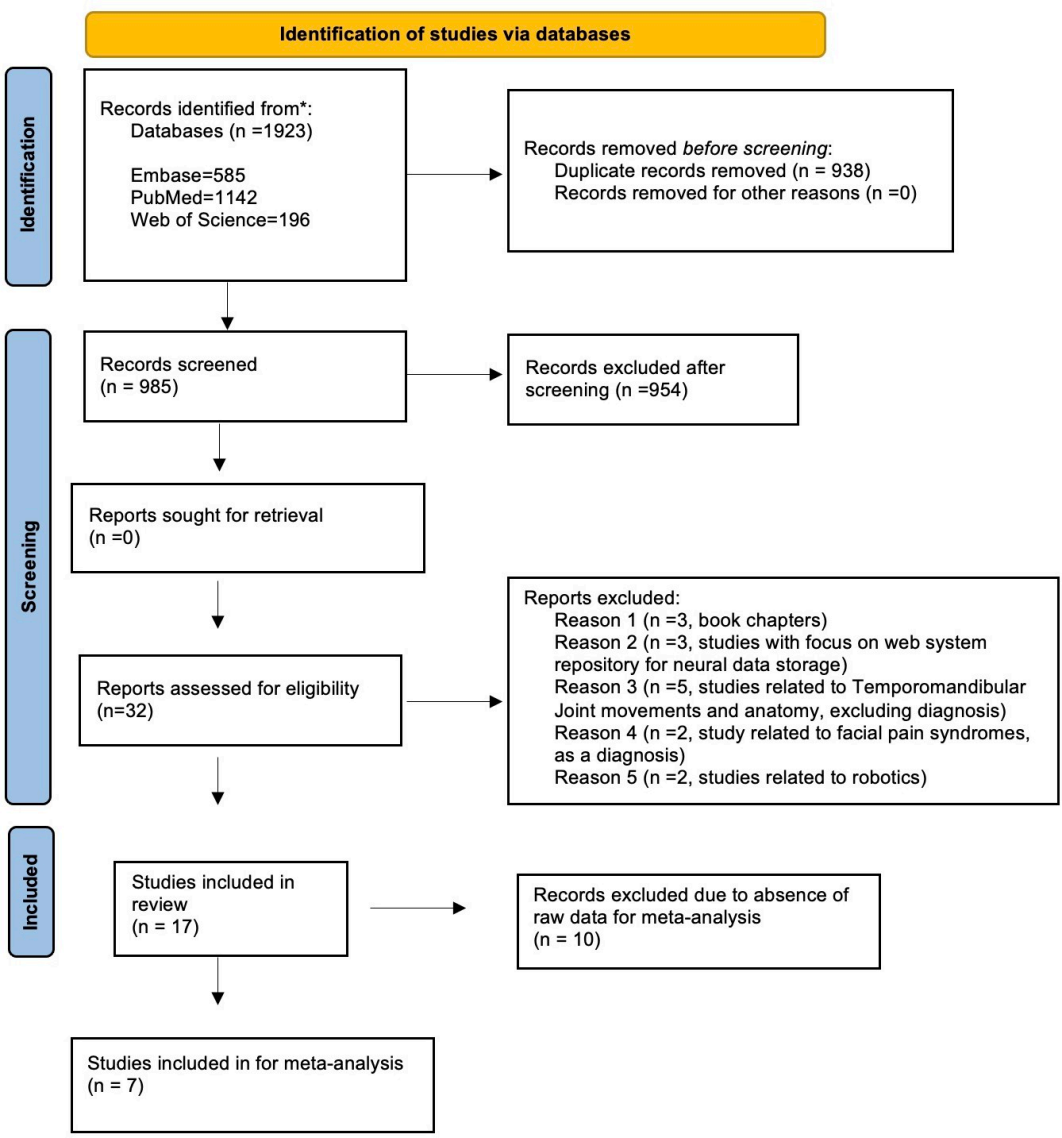
PRISMA flowchart for screening and identifying the included studies.

The initial database search yielded 1923 studies. After removing duplicate studies, 985 articles were screened for inclusion, of which 32 studies corresponded to TMD diagnosis using AI. However, 15 of these 32 articles were excluded due to various reasons, such as book chapters, studies with a focus on creating a web system repository for neural data storage, studies related to TMJ movement and anatomy, excluding diagnosis, studies related to facial pain syndrome as a differential diagnosis, and studies related to robotics (S3 Table). Finally, 17 articles met our eligibility criteria and were included in this systematic review (Fig 1).

### Risk of bias assessment of the included studies

**Fig 2:**
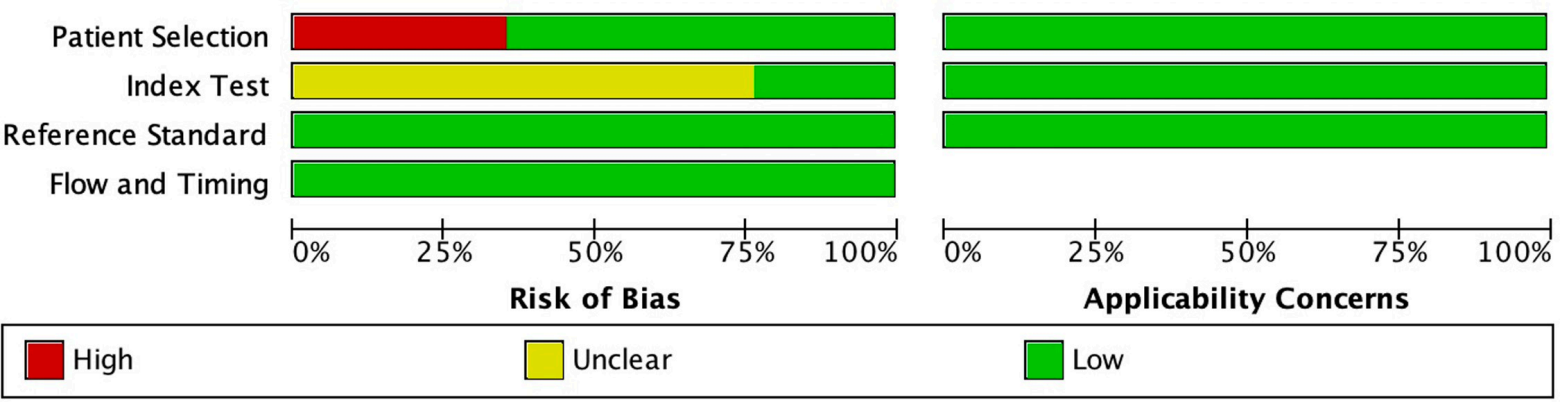
Quality assessment graph of included studies.

Fig 2 summarizes the study biases as high, low, or unclear. The patient selection bias potential was low in 11 out of 17 studies [17, 19, 21, 25–32] and high in 6 out of 17 studies [18, 20, 33–36]. A high risk of bias in patient selection was present due to the inclusion of case-control studies. However, the applicability concerns for patient selection were assessed as low for these studies because selection bias was overcome using case-control matching. Regarding the reference test and flow and timing domains, 17 out of 17 studies were considered to have a low degree of bias and low degree of applicability (S1 Fig). Index test was reported unclear for 13 out of 17 studies due to a lack of information on threshold values.

### Certainty of evidence assessment of the included studies

Of the 7 studies considered for meta-analysis, 2 studies had invalid outcomes for the test of diagnostic accuracy. Therefore, 5 studies were included for the GRADE analysis [17, 18, 28, 33, 34]. According to the GRADE, the Risk of bias was considered serious as it was high for three studies [18, 33, 34]. The factor of imprecision was considered very serious because the number of subjects was less than 1000 [17, 18, 33, 34]. Therefore, the certainty of evidence was concluded as very low (S4 Table).

### Characteristics of the included studies

The types of studies included were mostly retrospective studies [17, 20, 21, 25, 26, 28, 30–33], 3 case-control studies [18, 19, 29, 34], 2 case-control cohort studies [35, 36], and 1 pilot study [27]. Table 3 shows the characteristics of the included studies.

**Table 3:** Characteristics of the included studies.

Author, Year	Sample description (age in years and/ or sex)	Study objective	Type of Data used	Algorithms used	TMD subtype studied*	Criteria for diagnosing TMD subtype	Dataset size	Features used for training	Results/Performance
Radke et al., 2003 [29]	-	Artificial neural network for detection of normal TMJs and non-reducing displaced disks	Medical records	ANN	Internal derangement	medical history, clinical examination findings, joint vibration analysis findings, electromyographic findings, and using tomographic x-rays	Training set: 34 Testing set: 34	Incisal chewing patterns	a) Specificity: 100% b) Sensitivity: 91.8% c) Accuracy: 86.8%
Ghodsi et al.,2007 [19]	-	Automatic facial pattern classification between individuals with TMD and healthy individuals	High-resolution video camera	SVM	TMD (no subtype)	clinical examination findings	Mandible movements		Lyapunov exponent (λ_1_) larger for individuals with TMD than those for healthy subjects
Bas et al., 2012 [25]	-	Use of ANN for diagnosis of TMJ ID and normal joints	Medical records	ANN	Internal derangement	Patient histories and clinical symptoms, according to RDC/TMD*	Training set: 161 Testing set: 58	Clicking, joint sounds, and jaw deviation	Unilateral ADDwR a) Sensitivity: 80% b) Specificity: 95% Unilateral ADDwoR a) Sensitivity: 69% b) Specificity: 91% Bilateral ADDwR a) Sensitivity: 100% b) Specificity: 89% One side ADDwR, other side ADDwoR a) Sensitivity: 44% b) Specificity: 93%
Iwasaki, 2015 [21]	1. **Age**: a) Age Range:11–86 b) Average age:39.5 2.**Sex**: 54 males, 241 females	BBN application to MRI for diagnosis of TMDs	MRI	ANN; Bayesian belief network path condition, Greedy search-and-score, Bayesian information criterion, Chow–Liu tree, Rebane–Pearl poly tree, Naïve Bayes	Internal derangement and TMJOA	RDC/TMD or defined by the author (bony changes and disc displacement)	-	Disc displacement and bony changes within TMJ	Accuracy Model: 99% MRI, bony abnormalities: 60%–100% MRI, disc position: 73%–85%
Haghnegahdar et al., 2016 [26]	-	Local binary patterns for assessment of TMDs	CBCT	Random forest, Naïve Bayes, SVM, KNN, Local binary pattern, Histogram of oriented gradients	TMJOA	clinical examination findings	Training set: 132 Testing set: 132	Condylar shape	KNN a) Accuracy: 92% b) Sensitivity: 94% c) Specificity: 90% SVM a) Accuracy: 84% b) Sensitivity: 84% c) Specificity: 85% Naïve Bayes a) Accuracy: 75% b) Sensitivity: 78% c) Specificity: 73% Random forest a) Accuracy: 73% b) Sensitivity: 75% c) Specificity: 73%
de Dumast et al., 2018 [20]	-	Deep neural network to assess shape changes in TMJOA	CBCT	CNN	TMJOA	morphological variability in radiographs	268 TMJs	Condylar shape	Accuracy Training data: 93% Testing data: 95%
de Dumast et al., 2018 [33]	Mean Age a) TMJOA: 39.9± 11.7 b) controls: 39.4 ±15.4	web-based system for neural network classification of TMJOA	CBCT	CNN, PCA	TMJOA	medical history, clinical examination findings	Training set: 259 Testing set: 34	Serum and salivary biomarkers, condylar shape	PCA a) Pain variables > 82% b) Protein levels in plasma and saliva > 99%
Nam et al., 2018 [31]	1. **Age**: Mean Age a) TMD: 31.2 ± 15.8 b) TMD mimicking: 39.5 ± 23.2 2. **Sex**: 61 males, 229 females	NLP to differentiate TMD and TMD mimicking conditions	Medical Records	NLP	TMD (no subtype)	Medical records, RDC/TMD	-	Mouth opening	The goodness-of-fit of the model: 0.643 a) Accuracy: 96.6% b) Sensitivity: 69.0% c) Specificity: 99.3% d) Positive-predictive value: 90.9% e) Negative-predictive value: 97.0%
Ribera et al., 2019 [18]	Mean Age: 39.9± 11.7	Deep neural network to assess bony changes in TMJOA	CBCT	CNN	TMJOA	morphological variability in radiographs	Training set: 259 Testing set: 34	Condylar shape	Accuracy 47% of exact classification (91% for an error of +/–one group)
Shoukri et al., 2019 [34]	Mean Age a) symptomatic: 39.9± 11.7 b) controls: 39.4 ±15.4	Test correlations of biomarkers of condylar morphology and find deep neural network to assess bony changes in TMJOA	hr-CBCT	CNN	TMJOA	Clinical examination findings and radiographic diagnosis based on DC/TMD	Training set: 259 Testing set: 34	Articular fossa and condyle	Predictive analytics of neural network staging of TMJ OA (compared to clinicians’ consensus) showing degree of conformity. Training data: 73.5% Testing data: 91.2%
Bianchi et al., 2020 [35]	1.**Age** Age Range: 21–70 2.**Sex**: a) CG-7 males, 39 females b) TMJOA-7 males, 39 females	Diagnosis of TMJOA using biomarkers and machine learning	hr-CBCT	Light gradient boosting machine, XGBoost	TMJOA	DC/TMD	-	Radiomics and biomolecular variables, condylar shape	Accuracy: 0.823 AUC: 0.870 F1 score: 0.823
Bianchi et al., 2020 [36]	1.**Age** a) Age range-21-70 b) Mean age TMJOA-: 40.2 ±13.1 controls: 36 ±11.4 2.**Sex**: a) CG-6 males, 33 females b) TMJOA-7 males, 38 females	Diagnosis of TMJOA using quantitative bone imaging biomarkers	hr-CBCT	GLCM and GLRLM	TMJOA	DC/TMD	Control group: 39 TMJOA group: 45	Radiomics and biomolecular variables, condylar shape	1. ROC curves for variables that presented significant differences between the TMJ OA and control groups 2. Prediction for energy and entropy: AUC > 0.7 3. AUC for all variables ranged from 0.62 to 0.71
Calil et al., 2020 [27]	1. **Age**: Age range: 18–50 2. **Sex**: a) CG- 5 males, 15 females b) MG-3 males, 7 females c) AG-4 males, 6 females	Analysis of biomechanical features collected by an optoelectronic system to record jaw movements as a diagnostic tool for the evaluation of TMD.	Infrared camera with motion-tracking system	Random forest, Naïve Bayes, SVM, KNN	Myopathy and arthropathy	DC/TMD	-	Protrusion, lateral movements, opening and closing of mouth	KNN a) Precision: 93%–96% b) Accuracy: 95%–97% c) Sensitivity: 87%–97% d) Specificity: 94%–98% Random forest a) Precision: 66%–79% b) Accuracy: 79%–84% c) Sensitivity: 68%–79% d) Specificity: 79%–90% Naïve Bayes a) Precision: 63%–79% b) Accuracy: 80%–84% c) Sensitivity: 56%–84% d) Specificity: 76%–94% SVM a) Precision: 79% b) Accuracy: 79%–83% c) Sensitivity: 78%–88% d) Specificity: 77%–79%
Kim et al., 2020 [28]	1. **Age**: a) Age Range:20–60 b) Average age: 43.3 2. **Sex**: 700 males, 592 females	Automated detection of mandibular condyle using CNN and R-CNN	Panoramic radiograph, medical records	CNN	TMJOA	Patient history and clinical symptoms	1.**Detection**: Training set: 800 Testing set: 167 2.**Condyle discrimination:** Training set: 2066 Testing set: 518 3.**Classification**: Training set: 923 Testing set: 231	Articular fossa and condyle	Condyle validity classification (Model 2) a) Precision: 93% b) Recall: 83% c) F1 score: 93% Condyle abnormality classification (Model 3): best results shown by VGG16 Fine Tuning a) Accuracy: 84% b) Sensitivity: 54% c) Specificity: 94% d) AUC: 82%
Lee et al., 2020 [17]	1.**Age:** a) Age range:16–84 b) Mean age:39.5 ± 18.2 2. **Sex**: 84 males, 230 females	Automated assessment of TMJOA using CBCT images with AI	CBCT	SSD	TMJOA	RDC/TMD	Training set: 1757 Testing set: 300 Validation set:1757	Condylar shape	Accuracy: 0.86 Precision: 0.85 F1 score: 0.85 Recall: 0.84
Kim et al., 2021 [30]	1. **Age**: Median Age a) perforated group: 32 b) non-perforated group: 27 2. **Sex**: a) perforated group: 10 males, 120 females b) non-perforated group: 30 males, 138 females	Diagnosis of TMJ disc perforation using deep learning	MRI	MLP (ANN), Random forest	Disc perforation and TMJOA	Criteria defined by author based on MRI (disc shape, joint space, condylar changes)	-	Disc shape, condyle and fossa shape, joint space shape, and bone marrow	MLP showed highest performance a) AUC: 0.940 b) Sensitivity: 85.2% c) Specificity: 84.8% Random forest a) AUC: 0.918 b) Sensitivity: 96.3% c) Specificity: 75.8% Disc shape a) AUC: 0.791
Kreiner & Viloria, 2022 [32]	-	Diagnosis of TMD and orofacial pain using neural networks	Medical records	MLP (ANN)	Internal derangement	Criteria defined by author	-	Questionnaire consisting of symptom onset and description, quality of pain descriptors, pain intensity, time from onset, site & frequency of symptom, aggravating factors etc. comparing ability of MLP and dental practitioners to diagnose clinical cases	diagnostic accuracy of MLP superior to that of clinicians (p = .0072)

*TMD subtype is in accordance with the Diagnostic Criteria for Temporomandibular Disorders (DC/TMD) for Clinical and Research Applications [2]

ADDwoR, anterior disc displacement without reduction; ADDwR, anterior disc displacement with reduction; AG, arthropathy group; AI, artificial intelligence; ANN, Artificial neural network; AUC, area under the curve; BBN, Bayesian belief network; CBCT, cone-beam computed tomography; CG, control group; CNN, Convolutional neural networks; DC/TMD, Diagnostic Criteria for Temporomandibular Disorders; F1 score, harmonic mean of precision and recall; GLCM, gray-level co-occurrence matrix; GLRLM, gray-level run-length matrix; hr-CBCT, high resolution CBCT; IOU, intersection over union; KNN, K-nearest neighbors; MG, myopathy group; MLP, multilayer perception (artificial neural network, ANN); MRI, magnetic resonance imaging; PCA, principal component analysis; PCA, principal component analysis; RDC/TMD, Research Diagnostic Criteria for Temporomandibular Disorders; ROC, receiver operating characteristic; SSD, Single-Shot Detector; SVM, support vector machines; TMD, temporomandibular joint disorders; TMJ ID, Temporomandibular joint internal derangement; TMJOA, Temporomandibular joint osteoarthritis.

Sex distribution indicated higher number of female subjects than male subjects for most of the studies [17, 21, 27, 30, 31, 35, 36]. Image and nonimage data were used, and medical diagnostic imaging modalities, such as CBCT [17, 18, 20, 26, 33], high-resolution CBCT (HR-CBCT) [34–36], MRI [21, 33], and panoramic radiography [28] were used. Other types of image data included infrared cameras with a motion-tracking system [27] and high-resolution video cameras [19]. Nonimage data included medical records, such as patients’ symptoms [25, 29, 31, 32]. The most frequently used method was convolutional neural networks (CNNs; 7 studies), followed by artificial neural networks (ANN; 5 studies), and decision trees (4 studies). Other techniques included Bayesian networks (3 studies), support vector machines (SVMs; 3 studies), K-nearest neighbors (KNNs; 2 studies), and natural language processing (NLP; 1 study). Some studies used several machine-learning algorithms and compared the results.

### Meta-analysis

**Fig 3:**
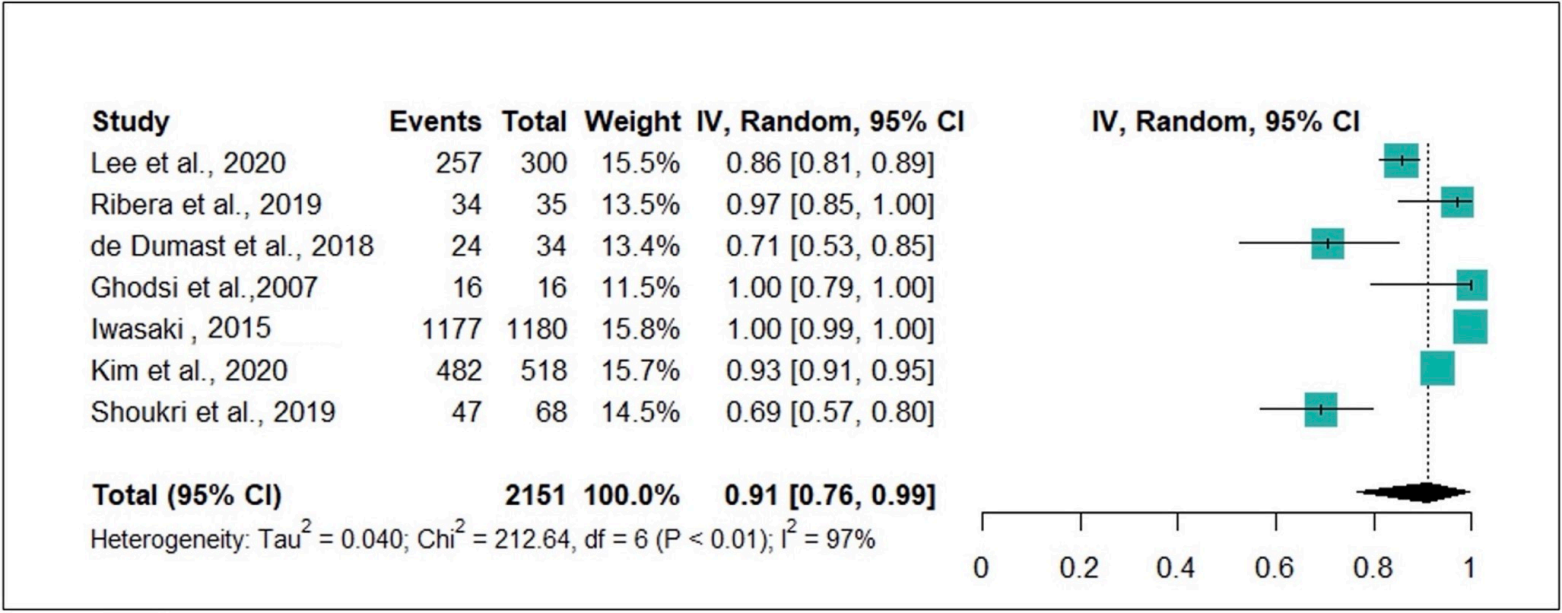
Meta-analysis of seven studies indicated by forest plot.

The diagnostic accuracy was 0.69–1.00, and the pooled accuracy was 0.91 (95% CI 0.76–0.99), I^2^ = 97% (95% CI 0.96–0.98), *p* < 0.001 (Fig 3). The study with the lowest accuracy had multiple classes of condylar shape in patients with DJD in which the classes represented varying degrees of condylar resorption and remodeling [33].

## Discussion

Diagnosis of TMDs can be complex as patients present with various symptoms according to subtypes, thus requiring clinical expertise. Various studies have diagnosed TMDs using AI to facilitate diagnosis and support clinical decisions. However, the accuracy of the developed models varied greatly depending on the type of data used, dataset size, and algorithms used for developing the model.

Among the subtypes of the TMDs, TMJOA was found to be the most studied type of TMD in this systematic literature review. One of the possible reasons is that TMJOA is an advanced form of disease that occurs after disc displacement, and it has a significant effect on occlusion and facial appearance. Deep-learning algorithms were used to diagnose TMJOA by detecting the changes in the condyle shape using CBCT images [18, 20, 33]. Lee et al. developed an automated diagnostic tool for detecting TMJOA based on the Diagnostic Criteria for TMDs [17]. Kim et al. used panoramic radiographs to automatically detect the condyles and classify osteoarthritis [28]. Although panoramic radiographs are not considered the standard imaging technique in the diagnosis of TMJOA [4], the AI model showed accuracy, sensitivity, and specificity of 0.84, 0.54, and 0.94, respectively, for diagnosing bony abnormality [28]. Machine-learning methods were used to examine correlations between the biomarkers, and condylar shape changes were investigated to increase diagnostic sensitivity [34–36]. Radiomics features were extracted from high-resolution CBCT scans to detect early bony changes [35, 36].

All studies on TMJOA used image data to analyze mandibular condyle shapes [17–20, 28–31, 33–36], and CBCT was the most commonly used imaging modality. Accurate assessment of bony changes is possible using CBCT; thus, it is considered the gold standard for TMJOA [37]. HR-CBCT scans at a submillimeter resolution with voxel size as low as 80 μm [38]. Compared with micro-CT, it allows observing subtle changes in the trabecular pattern of the condyle [35, 39]. The accuracy of the AI models used in these studies ranged from 80% to 90%, indicating their high reliability. These results are similar to the conventional studies involving human experts to diagnose TMJOA using CBCT [40, 41]. MRI was the most frequently used imaging method for the diagnosis of internal derangements and disc perforations [21, 30]. Other data include jaw movement records [27]. Bas et al. used clinical symptoms and diagnoses to predict the subtypes of internal derangements using ANNs [25]. We provide a brief explanation of techniques used in each study below.

ANN is a popular AI model that includes one input layer, two or three hidden layers, and one output layer. ANN training begins by randomly assigning weights as small numbers near 0 and iterating the feedforward and backpropagation algorithms until certain criteria are met to accurately predict the final output [42].

Deep learning is a subgroup of ANNs that involves many hidden layers. CNNs are a type of deep learning algorithms that have been developed for image data analysis. CNNs can be used for medical image analysis by performing tasks such as classification, which identifies input image data as pretrained classes (such as disease or normal), detection, which locates the region of interest (i.e. abnormal area), and segmentation, which identifies regions of interest as pixel-wise boundaries [43–45].

Decision trees are popular tools that present results in a tree structure that can be easily interpreted, are less time-consuming, and can help understand the interactions among different features [46]. Decision tree algorithms were used by four studies in various forms, such as random forest [26, 27, 30], light gradient boosting machine, and XGBoost [35].

Bayesian networks are a group of techniques connecting statistics and machine learning applicable to complex systems, which can leverage smaller data sizes compared with other machine-learning algorithms [47]. Further, large probability distributions can be compactly represented using Bayesian networks [48]. They comprise factorizing a probability distribution and a corresponding directed acrylic graph (DAG). The DAG presents a cause–effect relationship among nodes [21, 48]. Bayesian networks have many forms, including naïve Bayes (supervised classification) [45], greedy search-and-score [21], and Bayesian belief network path condition [21].

SVMs have been recently developed and are useful techniques in pattern recognition and classification studies [49]. Algorithm consideration, i.e., selecting a kernel/learning function, made in advance, can improve the performance of SVMs. This technique involves the nonlinear mapping of input vectors in a high-dimensional feature space to construct a linear decision surface [49].

KNN is one of the simplest classification methods wherein the samples are divided into training and testing groups. Training is performed with known labels, following which test samples are predicted using the learned model. The training and testing data need not be identical for KNN [50].

NLP is a subfield of AI that is used to decode human language into computer language [31]. Hospital data in the form of clinical history, radiology reports, and physical examination findings are available from clinical databases; these can be interpreted with computational linguistics using AI-assisted NLP systems. Free text can be organized into structured data [31, 51], which reduces labor-intensive and error-prone administrative demands.

Feature extraction techniques such as gray-level co-occurrence matrix, gray-level run-length matrix [36], local binary patterns [26], and histograms of oriented gradients [26] are used as image-processing techniques to automatically analyze texture, shape, and color changes within images. Feature selection is an important step in classification [52]. Different feature extraction algorithms can be sequentially applied to extract feature matrices for individual images. Following this method, feature matrix classification is performed using algorithms, such as SVM and KNN [52]. Principal component analysis (PCA) is a mathematical algorithm used to identify variations in data that simultaneously reduces their dimensionality, creating sample plotting, and identifying similarities and differences within a group of simple tasks [53].

Regarding the risk of bias assessment, this study used the QUADAS-2 tool recommended for systematic reviews of diagnostic accuracy by the Agency for Healthcare Research and Quality, Cochrane Collaboration [54]. We could have used the Cochrane tool for Risk Of Bias due to Missing Evidence in a synthesis. However, this tool was intended for risk of bias assessment for the meta-analyses of the effects of interventions [55]. Some of the included studies showed a high risk of bias in the patient selection domain because they were case-control studies. Other domains showed a low risk of bias and low risk of applicability concerns for all included studies.

Regardless of the possible risk of patient selection bias, most of the included studies reported high performance of the AI models showing a pooled accuracy of 0.91. However, there was a concern about the quality of evidence due to the small number of subjects included in the studies. Moreover, apart from the quality of the evidence, most studies lacked robust validation mechanisms. Validation, i.e., model performance evaluation, may be evaluated using data used for model development (internal) or from separate data that is not used for model development (external) [56]. Crossvalidation or validating from similar data sources may introduce accuracy bias [57]. External validation mechanisms, such as cohort studies, data collection from various institutions, prospective data [58], and data from different sites [56], are needed to improve the accuracy, quality, and generalizability of AI models.

Accuracy of traditional diagnostic tools for TMDs varies greatly. A systematic review on the diagnostic accuracy of clinical diagnostic tests and signs of TMD reported sensitivity and specificity of 2–89% and 14–97%, respectively [59]. The diagnostic accuracy varied according to the disease subtype and diagnostic test and signs used. In contrast, medical imaging modalities such as CT and MRI, which are regarded as gold standards for diagnosis of osteoarthritis and internal derangement, respectively, have shown a high examiner reliability [60]. Latest AI technologies have been introduced to support clinicians in diagnosing TMDs using various types of data, such as medical diagnostic images, video images, radiomics features, jaw movement tracking, electronic medical records (EMR), and biomarkers. These may contribute to the increased diagnostic accuracy.

This study has a few limitations. Most of the included studies have reported the model performance in terms of sensitivity, specificity, accuracy, recall, and R1. However, they did not provide raw data for meta-analysis of sensitivity and specificity, except for one study [14]. Therefore, only accuracy could be calculated in the meta-analysis. Additionally, the accuracies of the included studies showed high heterogeneity because the AI algorithms were developed for different TMD subtypes, thus the number of classes in the output and the criteria for accurate prediction varied among studies. Another limitation is that the study protocol was not registered in PROSPERO, and the transparency of this study could be affected. Lastly, we omitted abstracts and conference proceedings in our review and only used English articles selected from major databases, which collectively may exclude relevant studies published in other languages.

## Conclusions

The results of this study suggest that AI algorithms developed for automated TMD diagnosis can be used as a decision support tool for clinicians. In addition to the medical diagnostic images, various input data types, such as EMR, biomarkers, and radiomics features may help increase the diagnostic accuracy of TMDs. However, a high risk of bias in patient selection was present due to the inclusion of case-control studies. Most of the studies used a small training dataset and lacked external validation. Additionally, a significant heterogeneity was observed among the studies included for meta-analysis of diagnostic accuracy. The certainty of evidence was concluded as very low. Further studies with a larger dataset to prevent overfitting and ensure generalizability of developed models are warranted.

## Supporting information

S1 Fig Quality assessment (QUADAS-2) summary table for individual studies. (TIF)

S1 Table PRISMA 2020 for abstracts checklist. (DOCX)

S2 Table PRISMA 2020 checklist. (DOCX)

S3 Table List of excluded studies. (DOCX)

S4 Table GRADE assessment of the level of evidence for all included studies. (DOCX)
