# Supplementary figures and images for: Diagnosis of temporomandibular disorders using artificial intelligence technologies: A systematic review and meta-analysis
Source: PLoS One. 2022 Aug 18;17(8):e0272715. doi: 10.1371/journal.pone.0272715 (PMC9387829; doi:10.1371/journal.pone.0272715)

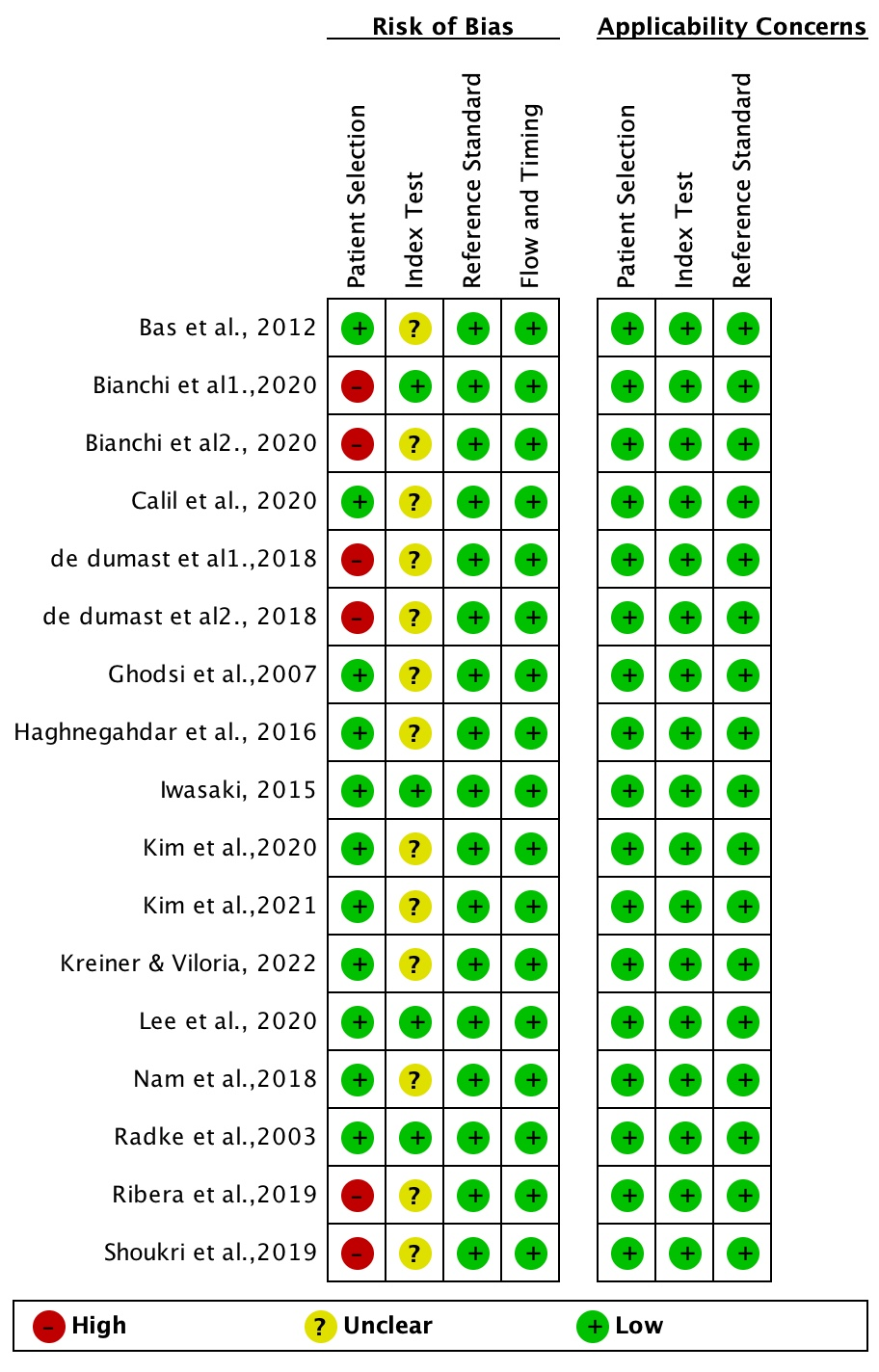

Supplement: S1 Fig — (TIF) [file pone.0272715.s001.tif]
