# Supplementary material for: Diagnosis of temporomandibular disorders using artificial intelligence technologies: A systematic review and meta-analysis
Source: PLoS One. 2022 Aug 18;17(8):e0272715. doi: 10.1371/journal.pone.0272715 (PMC9387829; doi:10.1371/journal.pone.0272715)
Supplement: S3 Table — (DOCX) [file pone.0272715.s004.docx]

**S3 Table. List of excluded studies**

| **Reasons for exclusion** | **Number of studies** | **List of excluded articles** |
| --- | --- | --- |
| Studies related to facial pain syndromes as a diagnosis | 2 | Harper DE, Shah Y, Ichesco E, Gerstner GE, Peltier SJ. Multivariate classification of pain-evoked brain activity in temporomandibular disorder. Pain Rep. 2016 Sep;1(3):e572 |
|  |  | McCartney S, Weltin M, Burchiel KJ. Use of an artificial neural network for diagnosis of facial pain syndromes: an update. Stereotact Funct Neurosurg. 2014;92(1):44-52 |
| Studies related to robotics and neural networks | 2 | Hiraiwa Y, Ariji Y, Kise Y, Sakuma S, Kurita K, Ariji E. Efficacy of massage treatment technique in masseter muscle hardness: robotic experimental approach. Cranio. 2013 Oct;31(4):291-9 |
|  |  | Ariji Y, Nakayama M, Nishiyama W, Ogi N, Sakuma S, Katsumata A, Kurita K, Ariji E. Potential clinical application of masseter and temporal muscle massage treatment using an oral rehabilitation robot in temporomandibular disorder patients with myofascial pain. Cranio. 2015 Oct;33(4):256-62 |
| Studies related to Temporomandibular Joint movements and anatomy, excluding diagnosis | 5 | Liu Y, Lu Y, Fan Y, Mao L. Tracking-based deep learning method for temporomandibular joint segmentation. Ann Transl Med. 2021 Mar;9(6):467. doi: 10.21037/atm-21-319 |
|  |  | Brosset S, Dumont M, Bianchi J, Ruellas A, Cevidanes L, Yatabe M, Goncalves J, Benavides E, Soki F, Paniagua B, Prieto J, Najarian K, Gryak J, Soroushmehr R. 3D Auto-Segmentation of Mandibular Condyles. Annu Int Conf IEEE Eng Med Biol Soc. 2020 Jul;2020:1270-1273 |
|  |  | Kwak GH, Kwak EJ, Song JM, Park HR, Jung YH, Cho BH, Hui P, Hwang JJ. Automatic mandibular canal detection using a deep convolutional neural network. Sci Rep. 2020 Mar 31;10(1):5711 |
|  |  | Lucena CV, Lacerda M, Caldas R, De Lima Neto FB, Rativa D. Mastication Evaluation With Unsupervised Learning: Using an Inertial Sensor-Based System. IEEE J Transl Eng Health Med. 2018 Apr 2;6:2100310 |
|  |  | Santos IC, Tavares JM, Mendes JG, Paulo MP. Acquisition and analysis of 3D mandibular movement using a device based on electromagnetic sensors and a neural network. J Med Eng Technol. 2009;33(6):437-41 |
| Studies with focus on web system repository for neural data storage | 3 | Michoud L, Huang C, Yatabe M, Ruellas A, Ioshida M, Paniagua B, Styner M, Gonçalves JR, Bianchi J, Cevidanes L, Prieto JC. A web-based system for statistical shape analysis in temporomandibular joint osteoarthritis. Proc SPIE Int Soc Opt Eng. 2019 Feb;10953:109530T |
|  |  | Paniagua B, Cevidanes L, Walker D, Zhu H, Guo R, Styner M. Clinical application of SPHARM-PDM to quantify temporomandibular joint osteoarthritis. Comput Med Imaging Graph. 2011 Jul;35(5):345-52 |
|  |  | Yatabe M, Prieto JC, Styner M, Zhu H, Ruellas AC, Paniagua B, Budin F, Benavides E, Shoukri B, Michoud L, Ribera N, Cevidanes L. 3D superimposition of craniofacial imaging-The utility of multicentre collaborations. Orthod Craniofac Res. 2019 May;22 Suppl 1(Suppl 1):213-220 |
| Book chapters | 3 | Choi E, Kim D, Lee JY, Park HK. Artificial intelligence in detecting temporomandibular joint osteoarthritis on orthopantomogram. Sci Rep. 2021 May 13;11(1):10246 |
|  |  | Sharma N, Gaffar Dar I, Kumar J, Khan A, Thakur A. Temporomandibular Joint Syndrome Prediction Using Neural Network. Lecture Notes in Electrical Engineering, Engineering Vibration, Communication and Information Processing, 2019. |
|  |  | Perpetuin D, Trippetti N, Cardone D, Breda L, D’Attilio M, Merla A. Detection of Temporomandibular Joint Disfunction in Juvenile Idiopathic Arthritis Through Infrared Thermal Imaging and a Machine Learning Procedure. 8th European Medical and Biological Engineering Conference, Springer, 2021. |
