# Supplementary material for: Diagnosis of temporomandibular disorders using artificial intelligence technologies: A systematic review and meta-analysis
Source: PLoS One. 2022 Aug 18;17(8):e0272715. doi: 10.1371/journal.pone.0272715 (PMC9387829; doi:10.1371/journal.pone.0272715)
Supplement: S4 Table — (DOCX) [file pone.0272715.s005.docx]

**S4 Table. GRADE assessment of the level of evidence for all included studies**

**Question**: Should AI methods be used to diagnose TMD in the general population?

| Outcome | № of studies (№ of patients) | Study design | Factors that may decrease certainty of evidence | | | | | Test accuracy CoE |
| --- | --- | --- | --- | --- | --- | --- | --- | --- |
|  |  |  | Risk of bias | Indirectness | Inconsistency | Imprecision | Publication bias |  |
| **True positives** (patients with TMD) | 5 studies 365 patients | cohort & case-control type studies | serious^a^ | not serious | not serious | very serious^b^ | none | ⨁◯◯◯ Very Low |
| **False negatives** (patients incorrectly classified as not having TMD) |  |  |  |  |  |  |  |  |
| **True negatives** (patients without TMD) | 5 studies 590 patients | cohort & case-control type studies | serious^a^ | not serious | not serious | very serious^b^ | none | ⨁◯◯◯ Very Low |
| **False positives** (patients incorrectly classified as having TMD) |  |  |  |  |  |  |  |  |

**Explanations**

a. Risk of bias high in some studies

b. less number of patients (less than 1000)

1. Pooled accuracy was calculated.

2. 5 studies were included for the GRADE analysis

*CoE, Certainty of Evidence
